# Minimally invasive coronary artery bypass grafting via a left lateral mini-thoracotomy in severe hemophilia A: a case report

**DOI:** 10.1186/s44215-026-00254-5

**Published:** 2026-04-01

**Authors:** Katsuhiro Hosoyama, Minami Yamada-Fujiwara, Kota Itagaki, Tatsuya Tago, Kentaro Yuda, Koki Ito, Yusuke Suzuki, Shintaro Katahira, Goro Takahashi, Kiichiro Kumagai, Yoshikatsu Saiki

**Affiliations:** 1 Division of Cardiovascular Surgery, Tohoku University Graduate School of Medicine, 1-1 Seiryo-machi, Aoba-ku, Sendai, Miyagi 980-8574 Japan; 2 Department of Hematology, Tohoku University Hospital, Sendai, Miyagi Japan

**Keywords:** Hemophilia A, Minimally invasive coronary artery bypass grafting, Factor VIII, Coronary artery disease, Case report

## Abstract

**Background:**

Life expectancy in patients with hemophilia A has increased owing to advances in factor VIII replacement, and the prevalence of age-related comorbidities such as coronary artery disease (CAD) is rising. However, coronary revascularization in severe hemophilia A remains challenging because both antithrombotic therapy and surgery can precipitate serious bleeding. We report a case of minimally invasive coronary artery bypass grafting (MICS-CABG) in a patient with severe hemophilia A, focusing on perioperative factor VIII management.

**Case presentation:**

A 50-year-old man (height 167 cm, weight 81 kg) with severe hemophilia A (baseline factor VIII activity < 1%) was followed at our institution. His bleeding history included mucosal and gastrointestinal hemorrhage requiring on-demand factor VIII replacement. He presented with exertional chest pain, and coronary angiography revealed single-vessel CAD involving the left anterior descending (LAD) and diagonal territory. Surgical revascularization was selected to avoid the need for long-term dual antiplatelet therapy after percutaneous coronary intervention involving an indwelling drug-eluting stent. Preoperative echocardiography showed preserved left ventricular function.

MICS-CABG via a left lateral mini-thoracotomy was performed, with the left internal thoracic artery (LITA) used as a sequential graft to the diagonal branch and LAD. Perioperative hemostasis was managed in collaboration with the hematologists using recombinant factor VIII. A preoperative bolus was followed by continuous infusion and scheduled dose adjustments based on serial factor VIII activity measurements. Total operative time was 5 h 32 min, and intraoperative blood loss was 407 mL without transfusion. No major bleeding or thrombotic events occurred. Postoperative coronary computed tomography confirmed excellent graft patency, and the patient was discharged without complications.

**Conclusions:**

Even in severe hemophilia A, MICS-CABG can be performed safely when combined with meticulous perioperative factor VIII replacement and activity monitoring. A minimally invasive surgical approach may help limit blood loss and transfusion requirements, and represents a useful revascularization option for selected patients with hemophilia and CAD.

**Supplementary Information:**

The online version contains supplementary material available at 10.1186/s44215-026-00254-5.

## Background

Hemophilia A is an X-linked recessive bleeding disorder caused by congenital deficiency or dysfunction of factor VIII. Disease severity is categorized by factor activity as severe (< 1%), moderate (1–5%), and mild (> 5–40%) [1]. Advances in recombinant factor VIII (rFVIII) concentrates and prophylactic replacement have markedly improved prognosis, with recent reports suggesting near-normal life expectancy in many patients with hemophilia [2].

Consequently, age-related cardiovascular diseases, including coronary artery disease (CAD), are increasingly observed in this population. Management of CAD in patients with hemophilia is complex because both percutaneous coronary intervention (PCI) and coronary artery bypass grafting (CABG) require peri-procedural antithrombotic therapy and carry a risk of severe bleeding. Contemporary reviews emphasize the need for individualized strategies and close collaboration between cardiologists, cardiac surgeons, and hematologists [3].

Several case reports and small series have demonstrated that CABG is feasible in patients with hemophilia when coagulation factor levels are carefully maintained perioperatively, although bleeding complications and transfusion are not uncommon [4, 5].

However, reports of minimally invasive coronary artery bypass grafting (MICS-CABG) in severe hemophilia A are scarce.

We report a case of severe hemophilia A complicated by exertional angina successfully treated with MICS-CABG under continuous factor VIII replacement, without blood transfusion. The case highlights practical aspects of perioperative management and the potential advantages of a minimally invasive approach.

## Case presentation

A 50-year-old man with severe hemophilia A (baseline factor VIII activity < 1%) was followed at our hospital. He had no history of prophylactic factor replacement and received only on-demand therapy. His bleeding history included oral bleeding after tooth extraction 7 years earlier, ankle joint bleeding after trauma 5 years earlier, upper gastrointestinal bleeding from reflux esophagitis 3 years earlier, and colonic polyp and diverticular bleeding within the preceding 2 years.

His past medical history included hypertension and syringomyelia under observation.

On presentation, he complained of exertional chest pain. Laboratory tests showed hemoglobin 13.9 g/dL, platelet count 178 × 10³/µL, prothrombin time–international normalized ratio 0.87, activated partial thromboplastin time 40.4s, and mildly elevated D-dimer(2.2 µg/mL). Baseline liver and renal function were normal.

Electrocardiography showed no significant ST–T changes, and echocardiography revealed preserved left ventricular systolic function (left ventricular ejection fraction 68%) without regional wall motion abnormalities or significant valvular disease. Coronary angiography demonstrated significant stenosis of the first diagonal branch (D1) and the left anterior descending (LAD) artery, consistent with single-vessel CAD. Given his high bleeding risk, long-term dual antiplatelet therapy after PCI was considered undesirable, and the heart team elected to perform surgical revascularization.

### Perioperative factor VIII management

Perioperative management was planned in collaboration with hematologists based on national guidelines for hemophilia care, with the goal of maintaining factor VIII activity at ≥ 100% during surgery and ≥ 50% in the early postoperative period [6].

**Fig. 1:**
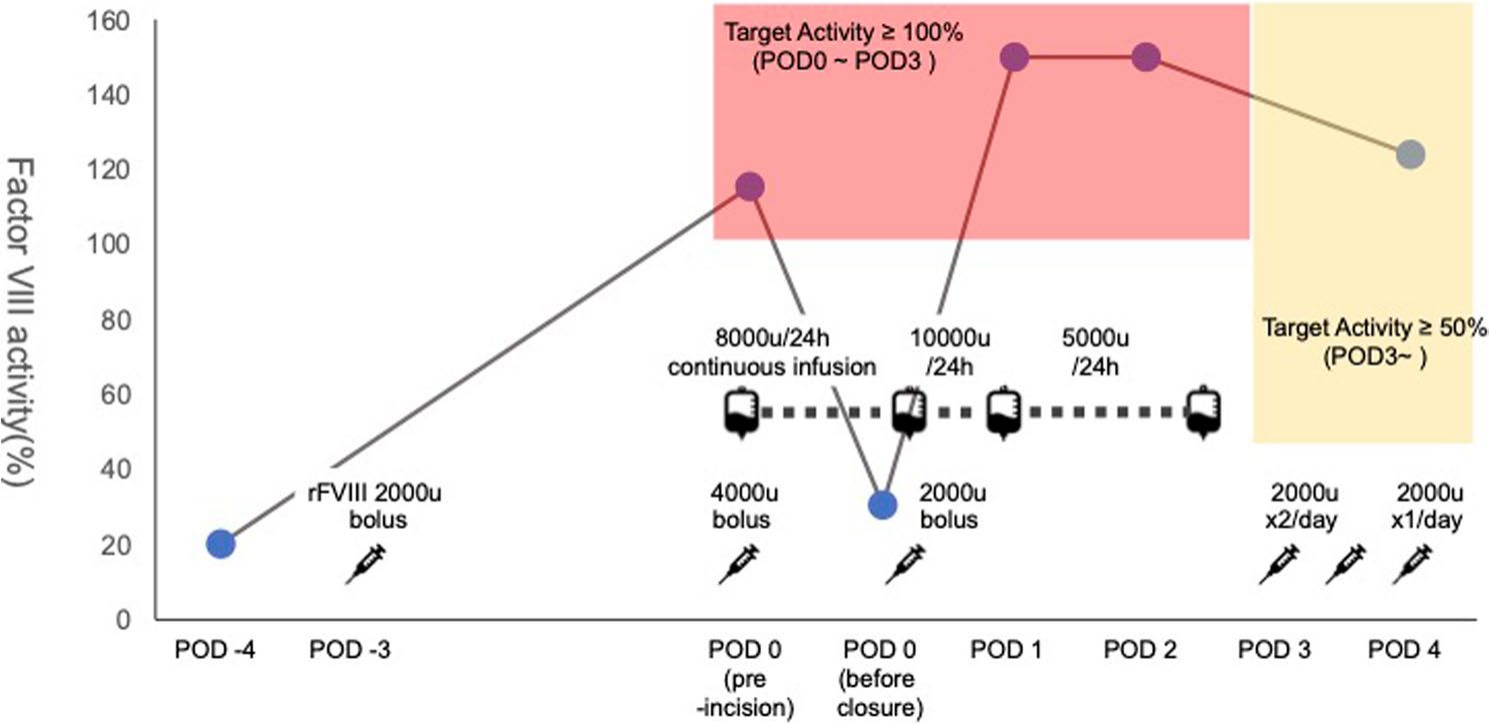
Perioperative rFVIII replacement and serial factor VIII activity. Bolus dosing and continuous infusion were used to target factor VIII activity of ≥100% intraoperatively and ≥50% in the early postoperative period. POD, postoperative day; rFVIII, recombinant factor VIII

As a substitute for the patient’s once-weekly emicizumab prophylaxis administered prior to admission, a bolus dose of rFVIII (Advate^®^, Takeda Pharmaceutical, Japan) 2,000 units was given three days before surgery (Fig. 1). On the day of surgery, an additional 4,000-unit bolus of rFVIII was administered, and surgery was initiated after confirming that factor VIII activity had increased to > 100% (140.1%). Systemic heparinization was performed according to our standard protocol (150U/kg), and the intraoperative activated clotting time was maintained at approximately 250s. Continuous infusion of rFVIII was initiated intraoperatively at an initial rate corresponding to 8,000 units/day. Immediately before completion of surgery, during the final hemostatic assessment, factor VIII activity was remeasured and found to be 34.6%. Accordingly, an additional 2,000-unit bolus was administered, and the continuous infusion rate was increased to 10,000 units/day.

On postoperative day (POD) 1, factor VIII activity was 204%, and chest tube drainage had subsided. Aspirin (100 mg once daily) was therefore initiated, and the continuous infusion rate was reduced to 5,000 units/day, which was maintained through POD 2 during the patient’s stay in the intensive care unit. After 72 h postoperatively, the target factor VIII activity was set at ≥ 50%. From POD 3, bolus dosing of rFVIII 2,000 units twice daily was administered, followed by once-daily bolus dosing from POD 4 onward.

### Surgical technique

MICS-CABG was performed through a left lateral mini-thoracotomy at the 5th intercostal space (Fig. S1-A). Single-lung ventilation was used to facilitate exposure. The left internal thoracic artery (LITA) was harvested under direct vision, with meticulous hemostasis along the harvesting bed.

The LITA was used as a sequential graft to the D1 and the LAD. A suction stabilizer was applied through the 6th intercostal space along the midaxillary line to obtain adequate exposure. Distal anastomoses were created using running 8 − 0 polypropylene sutures, performed sequentially from the D1 to the LAD. After completion of grafting, hemostasis was carefully checked at normotensive pressure.

Total operative time was 5 h 32 min, and total intraoperative blood loss was 407 mL. No allogeneic blood products were transfused. The patient was extubated in the operating room and transferred to the intensive care unit in stable condition.

### Postoperative course

Continuous infusion of factor VIII was maintained for three days postoperatively (POD 0–2), with gradual tapering based on factor VIII activity and clinical status. No mediastinal re-exploration was required, and no clinically significant hematomas or thrombotic events occurred. No allogeneic blood products were transfused intraoperatively or postoperatively.

Postoperative coronary computed tomography angiography demonstrated excellent patency of the sequential LITA–D1–LAD graft (Fig. S1-B). The patient was discharged uneventfully on postoperative day 10 and remained free from anginal symptoms during a 1.5-year follow-up period.

## Discussion and conclusions

The management of CAD in patients with hemophilia requires a careful balance between bleeding and thrombosis. Because dual antiplatelet therapy after PCI increases bleeding risk, CABG is often favored in patients with significant coronary stenosis when adequate factor replacement can be assured. However, conventional median sternotomy CABG itself is associated with substantial blood loss and transfusion requirements.

Several reports have shown that CABG in hemophilia A is feasible under rigorous perioperative factor VIII replacement, but variable rates of bleeding and transfusion have been described (Table 1). In the present case, we combined MICS-CABG with continuous factor VIII infusion and achieved successful revascularization without transfusion or major bleeding, despite underlying severe hemophilia A.

**Table 1:** Reported cases of CABG in patients with severe hemophilia A, including the present case

Author, year	Age (y)	Procedure	Factor VIII administration	Blood Transfusion	Complications
Present case, 2026	50	MICS, off-pump	Bolus + CI	No	No
Zwagg, 2024 [7]	80	On-pump	Bolus + CI	No	No
Rajasekhar, 2022 [8]	47	On-pump	Bolus	Yes	Re-exploration for bleeding
Misgav, 2017 [9]	64	On-pump	Bolus + CI	Yes	No
Mohammadzadeh, 2015 [10]	79	On-pump	Bolus	NR	No
Sprunck, 2012 [11]	38	Off-pump	Bolus + CI	No	No
Barillari, 2012 [12]	58	On-pump	Bolus + CI	No	No
Eren, 2006 [13]	62	On-pump	Bolus	NR	No

*CAGB *coronary artery bypass grafting, *MICS *minimally invasive cardiac surgery, *CI *continuous infusion, *NR *not reported

PCI is an established option for single-vessel CAD and has been reported as feasible in patients with hemophilia A [5, 14]. However, evidence specifically addressing patients with severe hemophilia A requiring dual antiplatelet therapy remains limited, and optimal antiplatelet strategies in this population have not been clearly established. In the present case, given the patient’s severe hemophilia A, prior bleeding history, and relatively young age (50 years), avoidance of dual antiplatelet therapy was considered advantageous from a long-term risk–benefit perspective. Because the LAD and D1 lesions were anatomically suitable for LITA-based revascularization, minimally invasive CABG was selected.

This case highlights three clinically important points. First, advantages of a minimally invasive approach: a left lateral mini-thoracotomy avoids full sternotomy and may reduce bone marrow and soft-tissue bleeding, which is particularly beneficial in patients with congenital bleeding disorders. A previous report demonstrated that blood transfusion requirements were significantly lower in MICS-CABG [15]. In MICS approaches, careful attention should be paid to potential bleeding from the stabilizer insertion site and the intercostal muscle layer at the mini-thoracotomy. In this case, meticulous soft-tissue handling and routine hemostatic techniques were sufficient to achieve stable hemostasis. Second, continuous infusion and close monitoring of factor VIII: real-time factor VIII activity testing is not universally available. In procedures requiring cardiopulmonary bypass, to avoid excessive activity and potential thrombotic risk, factor replacement may be limited to low-dose intermittent boluses, which can result in subtherapeutic levels and bleeding. When frequent measurements are feasible, continuous infusion allows tighter control within the therapeutic range and a more stable perioperative hemostatic profile. Third, importance of multidisciplinary planning: optimal outcomes require close collaboration among cardiac surgeons, anesthesiologists, hematologists, and intensive care physicians. Preoperative discussion of target factor levels, infusion protocols, and contingency plans for bleeding is essential.

In conclusion, MICS-CABG with continuous factor VIII replacement allowed safe and effective revascularization in a patient with severe hemophilia A and exertional angina. When expertise in both minimally invasive cardiac surgery and hemophilia management is available, this strategy may represent a valuable option for selected patients with hemophilia and CAD.

## Supplementary Information

Supplementary Material 1: Fig S1. (A) The mini-thoracotomy incision was made in the left 5th ICS, and the chest tube was inserted in the left 6th ICS, just inferior to the main incision. (B) Three-dimensional reconstructed computed tomography angiography showing the sequential LITA–D1–LAD graft. ICS, intercostal space; LITA, left internal thoracic artery; D1, first diagonal branch; LAD, left anterior descending artery.

## Data Availability

All data generated or analyzed during this study are included in this published article.

## Abbreviations

ACS: Acute coronary syndrome
CABG: Coronary artery bypass grafting
CAD: Coronary artery disease
D1: First diagonal branch
LAD: Left anterior descending artery
LITA: Left internal thoracic artery
MICS: Minimally invasive cardiac surgery
PCI: Percutaneous coronary intervention
POD: Postoperative day
FVIII: Recombinant factor VIII

## References

[1] Srivastava A, Santagostino E, Dougall A, Kitchen S, Sutherland M, Pipe SW et al. WFH Guidelines for the Management of Hemophilia, 3rd edition. Haemophilia. 2020;26(Suppl 6):1-158. 32744769 10.1111/hae.14046

[2] Hassan S, Monahan RC, Mauser-Bunschoten EP, van Vulpen LFD, Eikenboom J, Beckers EAM, et al. Mortality, life expectancy, and causes of death of persons with hemophilia in the Netherlands 2001–2018. J Thromb Haemost. 2021;19(3):645–53. 33217158 10.1111/jth.15182 PMC7986360

[3] Lin PS, Yao YT. Perioperative Management of Hemophilia A Patients Undergoing Cardiac Surgery: A Literature Review of Published Cases. J Cardiothorac Vasc Anesth. 2021;35(5):1341–50. 32723585 10.1053/j.jvca.2020.06.074

[4] Fogarty PF, Mancuso ME, Kasthuri R, Bidlingmaier C, Chitlur M, Gomez K, et al. Presentation and management of acute coronary syndromes among adult persons with haemophilia: results of an international, retrospective, 10-year survey. Haemophilia. 2015;21(5):589–97. 25689278 10.1111/hae.12652

[5] Cohen OC, Bertelli M, Manmathan G, Little C, Riddell A, Pollard D, et al. Challenges of antithrombotic therapy in the management of cardiovascular disease in patients with inherited bleeding disorders: A single-centre experience. Haemophilia. 2021;27(3):425–33. 33749973 10.1111/hae.14296

[6] Japanese Society on Thrombosis and Hemostasis. Guidelines for hemostatic treatment in patients with hemophilia without inhibitors (2013 revision). Tokyo: Japanese Society on Thrombosis and Hemostasis; 2013.

[7] Vander Zwaag S, Brose S, Fassl J. Combination of bolus and continuous infusion of factor VIII in a patient with severe hemophilia A undergoing on-pump coronary artery bypass graft surgery. Clin Case Rep. 2024;12(11):e9468. 39493790 10.1002/ccr3.9468 PMC11530351

[8] Rajasekhar A, Arnaoutakis GJ, Janelle GM, Harris N, Wynn T, Anderson RD, et al. Multidisciplinary Management of a Hemophilia A Patient Requiring Coronary Artery Bypass Graft Surgery. J Cardiothorac Vasc Anesth. 2022;36(2):534–8. 34895963 10.1053/j.jvca.2021.10.029

[9] Misgav M, Mandelbaum T, Kassif Y, Berkenstadt H, Tamarin I, Kenet G. Thromboelastography during coronary artery bypass grafting surgery of severe hemophilia A patient - the effect of heparin and protamine on factor VIII activity. Blood Coagul Fibrinolysis. 2017;28(4):329–33. 27273141 10.1097/MBC.0000000000000575

[10] Mohammadzadeh A, Babapoursaatlou B. Coronary surgery in an old patient with hemophilia A. Aging Clin Exp Res. 2015;27(1):83–4. 24789221 10.1007/s40520-014-0234-y

[11] Sprunck A, Maechel AL, Levy F, Faradji A, Steib A. Off-pump myocardial revascularization in a patient with haemophilia A: a case report and operative strategies. Haemophilia. 2012;18(1):e20–2. 21943209 10.1111/j.1365-2516.2011.02660.x

[12] Barillari G, Pasca S, Erice F, Livi U. Successful double bypass in a patient with severe hemophilia A: a case report. J Thromb Thrombolysis. 2012;33(2):193–6. 22183177 10.1007/s11239-011-0664-8

[13] Eren A, Friedl R, Hannekum A, Gulbins H. Cardiac surgery in a patient with haemophilia A. Thorac Cardiovasc Surg. 2006;54(3):212–4. 16639687 10.1055/s-2005-872859

[14] Fogarty PF, Mancuso ME, Kasthuri R, Bidlingmaier C, Chitlur M, Gomez K, et al. Presentation and management of acute coronary syndromes among adult persons with haemophilia: results of an international, retrospective, 10-year survey. Haemophilia. 2015;21(5):589–97. 25689278 10.1111/hae.12652

[15] Walton AJ, Pineda AM, Rogers L, Davierwala PM, Zwischenberger BA. Review of minimally invasive coronary artery bypass grafting. Eur J Cardiothorac Surg. 2025;67(5):ezaf160. 40434908 10.1093/ejcts/ezaf160

