# Supplementary figures and images for: Minimally invasive coronary artery bypass grafting via a left lateral mini-thoracotomy in severe hemophilia A: a case report
Source: Gen Thorac Cardiovasc Surg Cases. 2026 Apr 1;5:19. doi: 10.1186/s44215-026-00254-5 (PMC13169817; doi:10.1186/s44215-026-00254-5)

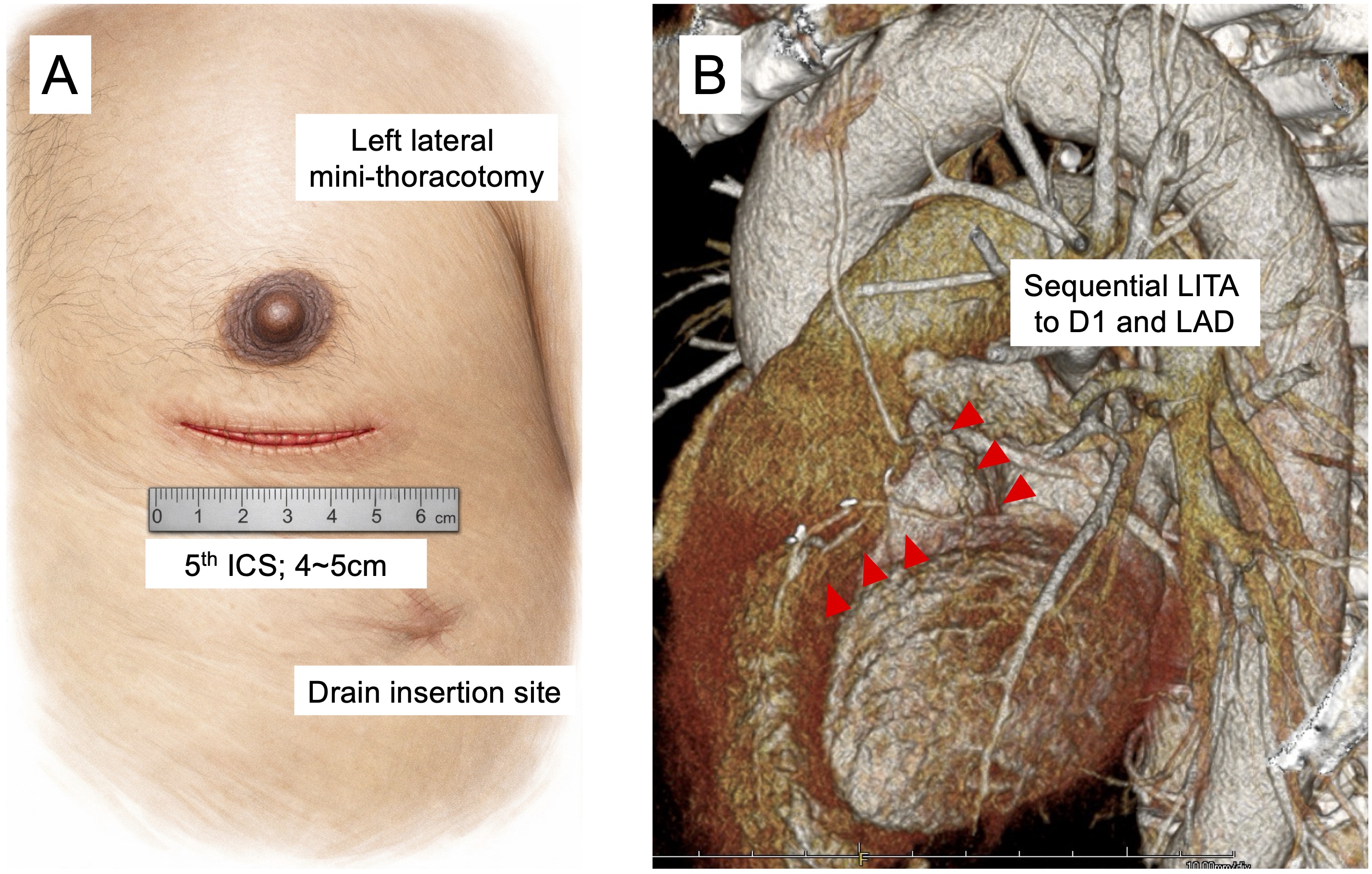

Supplement: Supplementary file 1 — Supplementary Material 1: Fig S1. (A) The mini-thoracotomy incision was made in the left 5th ICS, and the chest tube was inserted in the left 6th ICS, just inferior to the main incision. (B) Three-dimensional reconstructed computed tomography angiography showing the sequential LITA–D1–LAD graft. ICS, intercostal space; LITA, left internal thoracic artery; D1, first diagonal branch; LAD, left anterior descending artery. [file 44215_2026_254_MOESM1_ESM.jpg]
